# Inhibition of the oncogenic channel Kv10.1 by the antipsychotic drug penfluridol

**DOI:** 10.3389/fphar.2025.1655406

**Published:** 2025-09-03

**Authors:** Paulina León-Sánchez, Andrea Lizeth Cedillo-Hernández, María Luisa Durán-Pastén, Cesar Oliver Lara-Figueroa, Arturo Hernández-Cruz, Enoch Luis

**Affiliations:** ^1^ Laboratorio Nacional de Canalopatías, Instituto de Fisiología Celular, Universidad Nacional Autónoma de México, México City, México; ^2^ Departamento de Neuropatología Molecular, Instituto de Fisiología Celular, Universidad Nacional Autónoma de México, México City, México; ^3^ Investigador por México SECIHTI – Instituto de Fisiología Celular, Universidad Nacional Autónoma de México, México City, México

**Keywords:** penfluridol, Kv10.1 channel, carcinogenesis, oncogenic channel, antipsychotic

## Abstract

Drug repurposing is an increasingly recognized strategy in pharmaceutical development that focuses on identifying new therapeutic uses for already approved drugs, as an alternative to the time-consuming and costly process of developing new molecular entities. This approach has gained traction in oncology, especially in exploring the anticancer potential of non-oncologic agents. In recent years, the antipsychotic drug penfluridol (PNFL) has been identified as having antitumoral properties; however, the underlying mechanisms remain poorly understood. We hypothesized that PNFL may exert its effects by inhibiting the oncogenic potassium channel Kv10.1. Our results demonstrate that PNFL inhibits Kv10.1 activity and reduces cell migration in HEK-Kv10.1 cells. These findings suggest a novel mechanism that may contribute to the drug’s antitumoral effects.

## 1 Introduction

Over the past two decades, the voltage-gated potassium channel Kv10.1 has gained increasing relevance in oncology, as its expression is frequently high in a wide range of clinical cancer samples, while being absent in the corresponding non-tumoral tissues (Cázares-Ordoñez and Pardo, 2017; Luis et al., 2022a). Furthermore, the Kv10.1 channel has been shown to confer chemo- and radio-resistance to cells when overexpressed (Bai et al., 2013; Luis et al., 2022b); additionally, it has been considered a prognostic marker for certain cancers (Agarwal et al., 2010; Rodríguez-Rasgado et al., 2012; Ouadid-Ahidouch et al., 2016). All these have led to Kv10.1 being considered a promising therapeutic target for fighting cancer (Luis et al., 2022a). Currently, different pharmacological strategies (small molecules, antibodies, siRNA) to target Kv10.1 have shown positive results (Menéndez et al., 2012; Bernal-Ramos et al., 2017; Valdés-Abadía et al., 2019; Hartung et al., 2020; Toplak et al., 2021; 2022), decreasing some properties of cancer cells. However, finding drug candidates that selectively modulate Kv10.1 activity has been challenging.

In recent years, several reports have described that Penfluridol (PNFL), an oral antipsychotic agent used to treat psychotic disorders (Soares and Silva de Lima, 2006), has potential antitumoral properties (Tuan and Lee, 2019). For psychotic disorders, PNFL has been described as a dopamine receptor blocker (Tuan and Lee, 2019), while its anticancer activity appears to be related to the modulation of multiple targets, including ion channels (Gomora and Enyeart, 1999; Santi et al., 2002). PNFL, similar to other antipsychotic agents, has been associated with QT interval prolongation, indicating a potential interaction with cardiac ion channels, particularly the Kv11.1 channel (also known as the hERG channel) (Silvestre et al., 2014). In this context, PNFL has been characterized as a Kv11.1 channel blocker (Silvestre and Prous, 2007).

Kv10.1, along with Kv11.1 and seven other members, belongs to the KCNH gene family. Based on sequence similarities, the KCNH family is subdivided into three subfamilies: eag (Kv10.1 and Kv10.2), Herg (Kv11.1 to Kv11.3), and Elk (Kv12.1 to Kv12.3) (Gutman et al., 2005). All share sequence homology and common structural features. Notably, Kv10.1 and Kv11.1 exhibit a high degree of sequence homology, with 63% in the pore domain (Toplak et al., 2021). As a result, most Kv10.1 blockers also bind to Kv11.1 channels, which may pose cardiac risk (Barros et al., 2020; Toplak et al., 2021). Given the structural similarities and the known effect of PNFL on Kv11.1, we hypothesize that PNFL may modulate the activity of the oncogenic Kv10.1 channel.

In this study, we employ the FLIPR Membrane Potential Assay Kit from Molecular Devices, a method previously validated for identifying compounds that modulate Kv10.1 activity. This assay enables the rapid, non-invasive measurement of membrane potential changes, driven by the opening or closing of Kv10.1 channels (Gómez-Herrera et al., 2023). Here, we show that PNFL can modulate the fluorescence responses associated with changes in the membrane potential of HEK cells stably expressing the human Kv10.1 channel. Patch-clamp experiments supported that PNFL inhibits Kv10.1 activity in a concentration-dependent manner. Finally, the Kv10.1 modulation by PNFL affects the migration and morphology, but not the viability, of HEK-Kv10.1 cells. This study is the first report identifying penfluridol as a Kv10.1 inhibitor, revealing a previously unrecognized mechanism by which this antipsychotic drug may exert antitumoral effects.

## 2 Materials and methods

### 2.1 Cell culture

Cell lines were cultured as described by Loza-Huerta et al. (2021). Briefly, HEK293 wild-type cells (HEK-WT; CRL-1573, ATCC) and HEK293 cells stably expressing the human Kv10.1 potassium channel (HEK-Kv10.1) (generously provided by Dr. Walter Stühmer from the Max Planck Institute) were cultured in Dulbecco’s Modified Eagle Medium (DMEM) (12800-017, Gibco) supplemented with 10% fetal bovine serum (26140087, Gibco) and 1% Penicillin/Streptomycin (15140122, Gibco). HEK-Kv10.1 medium was supplemented with Zeocin (30 µg/mL) (R25001, Invitrogen) as a selection antibiotic. All cells were cultured at 37 °C in a 5% CO_2_ incubator.

### 2.2 High-throughput fluorescence assay

The FLIPR membrane potential (FMP) assay (R8034, Molecular Devices) was performed as described by Gómez-Herrera et al. (2023) and according to the manufacturer’s protocols. HEK-Kv10.1 (at a density of 20,000 cells/well in 100 µL of supplemented DMEM) were plated in flat-bottom 96-well plates (3599, Costar) and maintained for 24 h at 37 °C in a 5% CO_2_ incubator. Then, cells were loaded with a buffer containing FMP for 30 min at 37 °C in a 5% CO_2_ atmosphere. At this point, loperamide (LP) and penfluridol (PNFL) were mixed in the FMP buffer and pre-incubated in the selected wells. Based on our prior experience with small molecules targeting the Kv10.1 channel, including compounds such as loperamide (Loza-Huerta et al., 2021; Gómez-Herrera et al., 2023), 100 µM of PNFL served as a starting point for detecting functional effects. After pre-incubation, 96-well plates were moved to a FlexStation3 microplate reader (Molecular Devices) controlled by the SoftMax Pro 7 software. FLIPR^®^ dye was excited at 530 nm, and the emitted fluorescence was recorded at 565 nm. Fluorescence data were acquired every 2 s for 2 min: the first 20 s represent the basal fluorescence, and then, 50 µL (5x) of a high potassium solution was added (that resulted in a final K^+^ concentration of 60 mM), and the fluorescence signal was recorded for another 100 s. The fluorescence responses were normalized by F = F/F_0_, where F represents the fluorescence at any given time, and F_0_ is the mean basal fluorescence obtained in the first 20 s of recording. Once normalized, the amplitude was calculated as the difference between the basal fluorescence and the maximal amplitude at the end of the protocol. The first derivative of the fluorescence signal corresponds to the maximal rate of fluorescence increase.

### 2.3 Electrophysiological recording

HEK-Kv10.1 cells were plated on circular glass coverslips that had been previously treated with poly-l-lysine. Coverslips were transferred to a recording chamber (RC-26G, Warner Instruments), and whole-cell voltage-clamp recordings were performed at room temperature and under continuous perfusion (at a flow rate of 2 mL/min) with a standard bath solution. The standard bath solution contained (in mM): 137 NaCl, 5.4 KCl, 2 CaCl_2_, 1.3 MgCl_2_, 10 HEPES, and 10 D-glucose (300 mOsm/kg, pH 7.4 adjusted with NaOH). Intracellular patch-pipettes had resistances of 3–5 MΩ and were filled with an internal solution containing (in mM): 140 KCl, 1 MgCl_2_, 10 EGTA, 10 HEPES (300 mOsm/kg, pH 7.2 adjusted with KOH). Kv10.1 currents were recorded using a setup comprising a Multiclamp 700B (Molecular Devices), a Digidata 1550 (Molecular Devices), and controlled by pClamp10.6 software (Molecular Devices); data were filtered at 5 kHz and digitally sampled at 10 kHz. 40%–60% of the series resistance was electronically compensated. All patch-clamp recordings were made at a holding potential (Vh) of −70 mV. Kv10.1 currents were evoked with 250-ms-long voltage steps from −70 to +50 mV applied every 5 s. I-V curves were constructed using a protocol of 250-ms-long voltage steps from −100 mV to +50 mV in 10 mV increments. The maximal PNFL tested concentration (100 µM) was determined based on previous evidence from the FLIPR assay and was reduced gradually. Concentration-effect curves were fitted with the Hill equation using the Levenberg–Marquardt method implemented in Origin 2019 software: Inhibition = Bmax (C^n^/(IC50^n^ + C^n^)), where Bmax is the maximum block, IC50 is the concentration of half-maximal inhibition, C is the concentration of the molecules, and n is the Hill coefficient.

### 2.4 The wound healing assay

The migration rate was evaluated using the wound-healing assay. This assay was performed using the CytoSelect Wound Healing Assay Kit (#CBA-120, Cell Biolabs). The treated inserts generate a defined and reproducible gap in the wells. A 500 µL suspension of HEK-Kv10.1 cells (4 × 10^5^ cells/mL) was plated in wells pre-treated with Matrigel (CLS354234, Corning). Cells are cultured for 18–24 h until they form a monolayer around the insert. Then the insert is removed, leaving a space open, or a “wound field,” between the cells. Shortly after this period, cells were treated with mitomycin-C (12 µM) for 3 h to reduce the influence of proliferation on the migration behavior. Next, the culture media were replaced with media subjected to different treatments. Wound healing was evaluated by calculating the opening percentage through light field images acquired by the microscope ImageXpressXL (Molecular Devices). Wound size was measured for 0, 24, and 48 h. All treatments were replaced every 24 h.

### 2.5 Viability assay and morphology quantification

HEK-Kv10.1 and HEK-WT cells were plated at 1,500 cells per well in 96-well cell culture plates that had been previously treated with Matrigel (CLS354234, Corning). Cell viability was detected using a double fluorescence dye with Calcein Green-AM/Propidium Iodide (C34852/P1304MP, Invitrogen) to stain LIVE/DEAD cells, respectively, at 24 and 48 h post-treatment. Image acquisition was performed with the epifluorescence microscope ImageXpressXL (Molecular Devices) under ×40 magnification and two fluorescent channels to detect labeled live (green, ex/em: 495/515 nm) and dead (red, ex/em: 528/617 nm) cells. Cell viability quantification was made as follows:

$$\%\text{ cell viability}=\left(\text{live cells}/\left(\text{live cells}+\text{dead cells}\right)\right)*100$$

Additionally, we measure changes in the cell morphology of both cell lines under various treatments. Cells from three fields per well from three independent experiments were individually measured. The morphological change was measured by determining each cell’s major and minor axes and calculating the Morphological Cell Ratio (MCR), which is the ratio of the major axis versus the minor axis. To avoid reporting detached cells for both quantifications, LIVE/DEAD cells and MCR, the culture medium was replaced with PBS for image acquisition shortly after double fluorescence dye incubation.

### 2.6 Data analysis

Images were processed using Fiji (Schindelin et al., 2012). All data were analyzed using Origin 2019 (OriginLab, USA) and GraphPad Prism 8 (GraphPad Software, USA). Results are presented as mean ± SEM of at least three independent experiments. When two means were compared, statistical significance (P < 0.05) was assessed using Student’s t-test. For multiple comparisons, statistical significance (P < 0.05) was assessed using 1-way analysis of variance with the Dunnett *post hoc* test.

## 3 Results

### 3.1 PNFL decreases fluorescence signals in HEK-Kv10.1 cells

**FIGURE 1:**
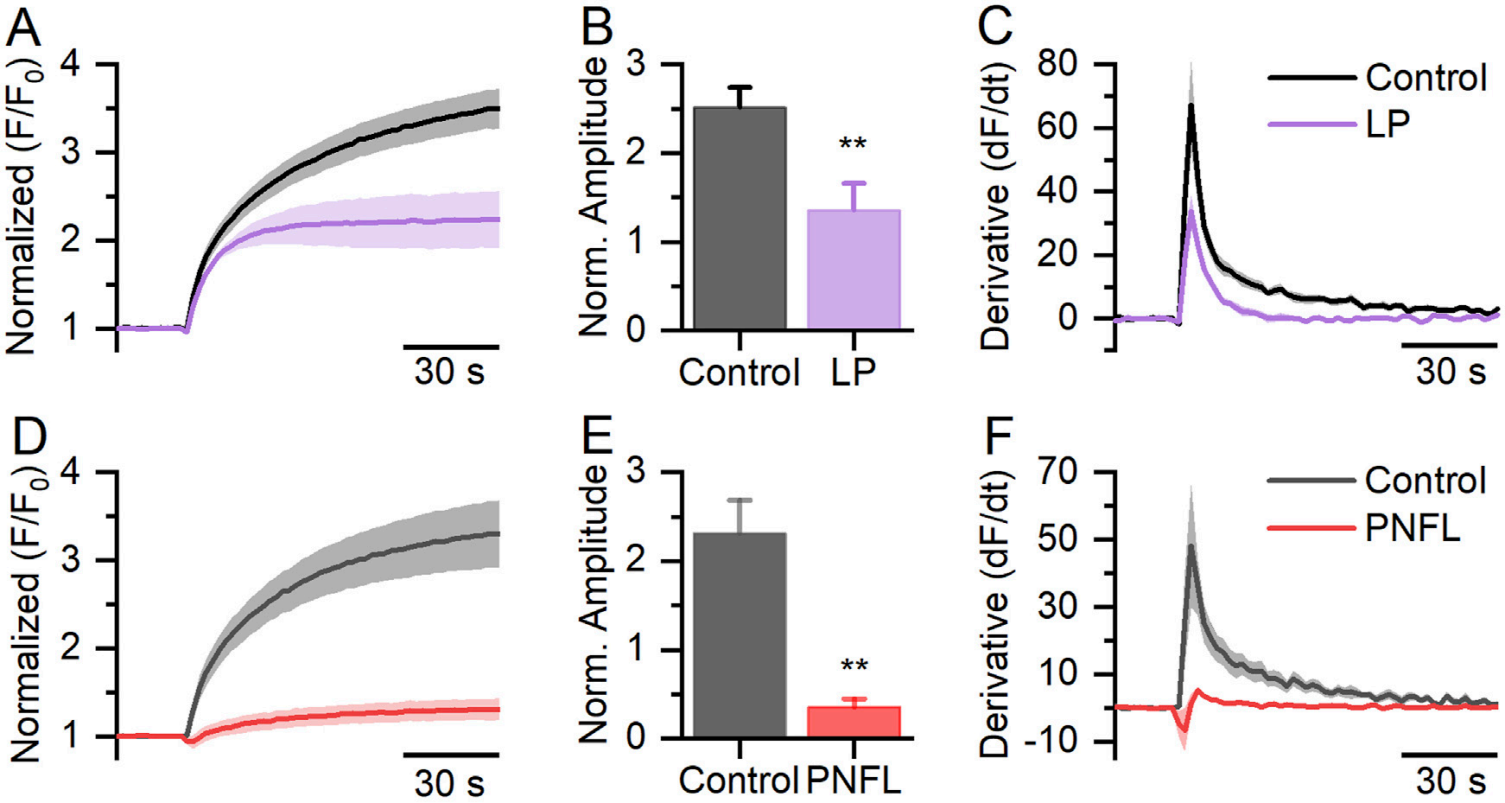
FLIPR^®^ membrane potential assay in HEK-Kv10.1 cells. **(A,D)** Fluorescence membrane potential recording in HEK-Kv10.1 cells in the presence of loperamide (LP, 100 µM) and Penfluridol (PNFL, 100 µM), respectively. The solid traces and faded colors show the mean ± SEM, respectively. **(B,E)** Summary graph of the fluorescence amplitude in HEK-Kv10.1 cells in the presence of LP and PNFL, respectively. Each bar represents the mean ± SEM of at least three independent experiments. **(C,F)** The first derivative of the fluorescent responses showed that LP and PNFL decrease the maximum velocity of change to the control conditions, respectively. ** for a P < 0.01, unpaired t-test.

PNFL has demonstrated anticancer effects (Tuan and Lee, 2019); however, the underlying molecular mechanisms of this effect are not well understood. Here, we hypothesize that one possible target of PNFL in cancer could be the oncogenic channel Kv10.1. We carried out a fluorescence membrane potential assay in HEK-Kv10.1 cells in the presence of PNFL. HEK-Kv10.1 cells produce a robust fluorescence response when stimulated with a high (60 mM) K+ solution (Figures 1A,D). As expected, the amplitude of the responses decreased by 46.1% in the presence of loperamide (100 μM), a well-known Kv10.1 inhibitor (Figures 1B-C). Interestingly, in the presence of PNFL (100 μM), the response amplitude was reduced by 84.7%, even more than LP (Figures 1D–F; P = 0.003, unpaired t-test). No changes in the signal fluorescence were observed in HEK-WT in the presence of PFNL, compared with the control condition (Supplementary Figures S1A,B).

### 3.2 PNFL inhibits Kv10.1 currents in a dose-dependent manner

**FIGURE 2:**
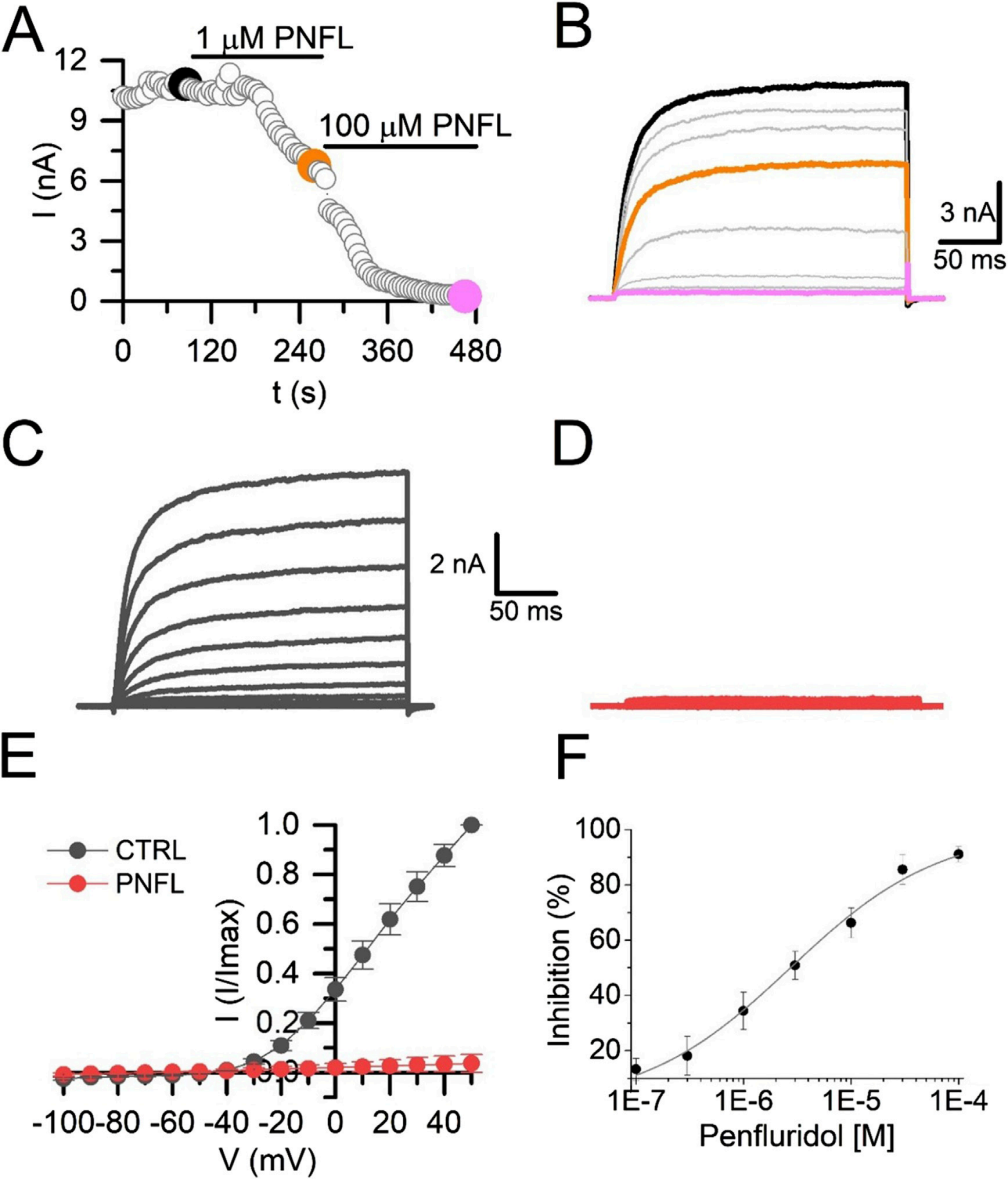
Penfluridol inhibits Kv10.1 currents. **(A)** Time course of the PNFL effect (1 and 100 µM) on the Kv10.1 currents measured at +50 mV. **(B)** Kv10.1-evoked currents in control (black) and in the presence of 1 µM (orange) and 100 µM (pink) of PNFL. Kv10.1 currents were evoked by a voltage step from −70 mV to +50 mV. **(C,D)** Family of Kv10.1 currents in the control (black) condition and the presence of 100 µM of PNFL (red), respectively. **(E)** IV curve relationship in the control (black) and in the presence of PNFL (100 μM; red). **(F)** Concentration-response curve of the inhibitory effect of PNFL on Kv10.1 activity. Each point represents n ≥ 3 cells. The solid line represents the fit to the Hill equation.

Next, we validated these fluorescence inhibitory results using whole-cell voltage-clamp recordings. We observed that PNFL reduced Kv10.1 currents in a dose-dependent manner; at 100 μM, the inhibitory effect was not reversible. PNFL (100 μM) produced an almost complete inhibition of Kv10.1 currents (96.3% ± 2.1%; n = 3; Figures 2A–E). The inhibitory effect of PNFL did not recover after washout (data not shown). Concentration-response data were fitted to a Hill equation, which yielded an IC_50_ of 2.7 ± 0.3 µM and a Hill coefficient of 0.55 ± 0.085 (Figure 2F). We also evaluated whether PNFL could modify the activity of the endogenous currents of HEK-WT cells. PNFL did not show a significant effect on currents measured at +50 mV, from 63.5 ± 10.7 pA in control to 42.4 ± 13.1 pA in the presence of 100 µM PNFL (n = 5; p = 0.68, paired t-test) (Supplementary Figures S1C,D).

### 3.3 PNFL reduces the migration associated with Kv10.1 overexpression

**FIGURE 3:**
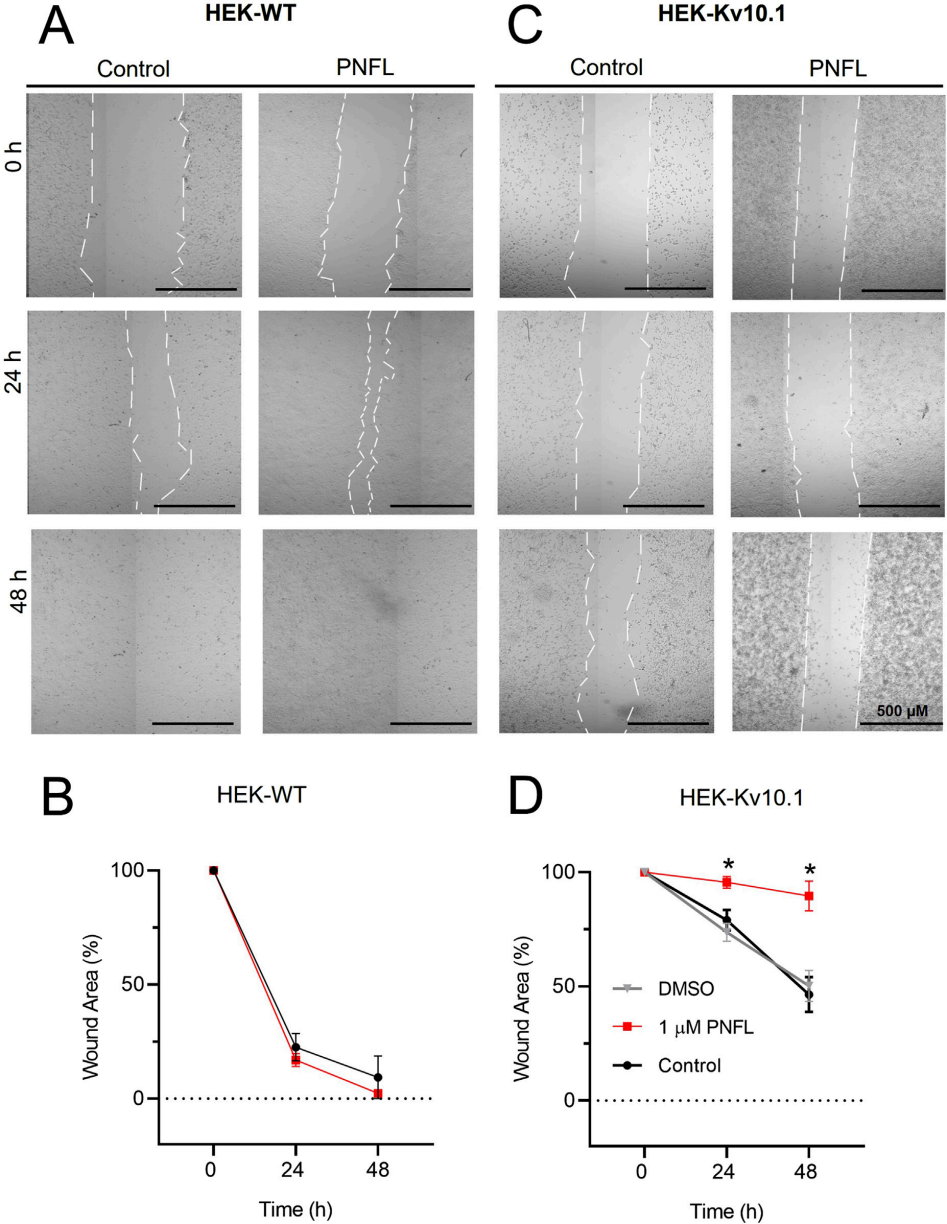
Penfluridol reduces the migration rate associated with Kv10.1 overexpression. **(A,C)** Representative cell migration assay at 0, 24, and 48 h of HEK-WT and HEK-Kv10.1 cells in control conditions and the presence of penfluridol, respectively. **(B,D)** Summary of the average percentage of wound area at 0, 24, and 48 h of HEK-WT and HEK-Kv10.1 cells in control and the presence of penfluridol, respectively. * for a P < 0.05, one-way ANOVA.

Kv10.1 channel activity has been described as necessary for migration in some cancer cells (Hammadi et al., 2012; Valdés-Abadía et al., 2019). For this reason, we performed the wound healing assay to evaluate whether PNFL reduces the migration rate associated with overexpression of the Kv10.1 channel. The migration rate was expressed as the percentage of area reduction or wound closure. Where 100% is the area at 0 h, which will decrease over time. We found that the migration rate of HEK-WT was not modified by the PNFL treatment (Figures 3A,B), and the wound area closed after 48 h. However, in HEK-Kv10.1 control condition the wound percentage was reduced to 74.7% ± 3.9% and 50.3% ± 3.9% at 24 and 48 h, respectively; in contrast to HEK-Kv10.1 cells exposed to PNFL (1 μM), the percentage of wound closure was lower, being 95.6% ± 2.5% and 89.6% ± 6.4% at 24 and 48 h, respectively (Figures 3C, D). These results indicate that PNFL (1 µM) significantly inhibits the migration of HEK-Kv10.1 cells (P = 0.0105, one-way ANOVA). No changes in the migration were observed in HEK-Kv10.1 in the presence of DMSO (0.1%), compared with the control condition.

### 3.4 PNFL does not affect cell viability

**FIGURE 4:**
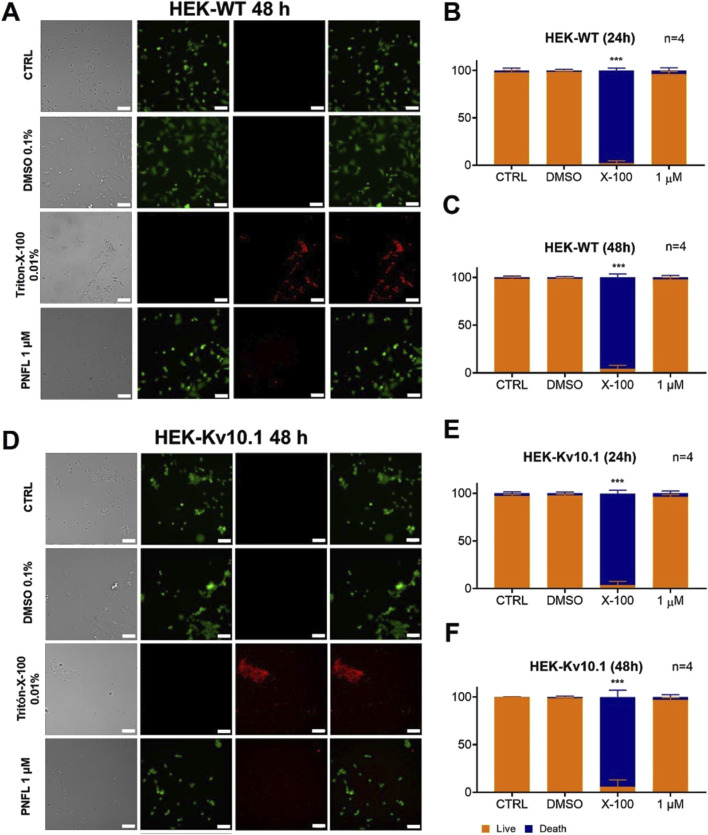
Penfluridol does not affect cell viability. **(A,D)** Representative fluorescence images of LIVE/DEAD cell viability assay in HEK-WT and HEK-Kv10.1 cells, respectively. Cells were loaded with Calcein Green-AM (Green) and Propidium Iodide (red) to stain LIVE/DEAD cells, respectively. Each column displays cells under transmitted light (left), calcein green fluorescence (second), propidium iodide fluorescence (third), and the superimposition of calcein green and propidium iodide (right). **(B,C)** Summary of the PNFL treatment on HEK-WT viability. **(E,F)** Summary of the PNFL treatment on HEK-Kv10.1 viability. *** for a P < 0.001, one-way ANOVA.

To verify that the effect of PNFL on the migration of HEK-Kv10.1 cells was not associated with a cytotoxic effect, cell viability assays were performed using double labeling with calcein-green and propidium iodide, which label live and dead cells, respectively. Neither HEK-WT nor HEK-Kv10.1 cells showed significant changes in cell viability in the presence of different concentrations of PNFL compared to the control condition (Figure 4). Treatment with Triton X-100 (0.1%) was used as a control for dead cells, significantly increasing cell death after its application (Figure 4).

### 3.5 PNFL modifies the morphology of HEK-Kv10.1 cells

**FIGURE 5:**
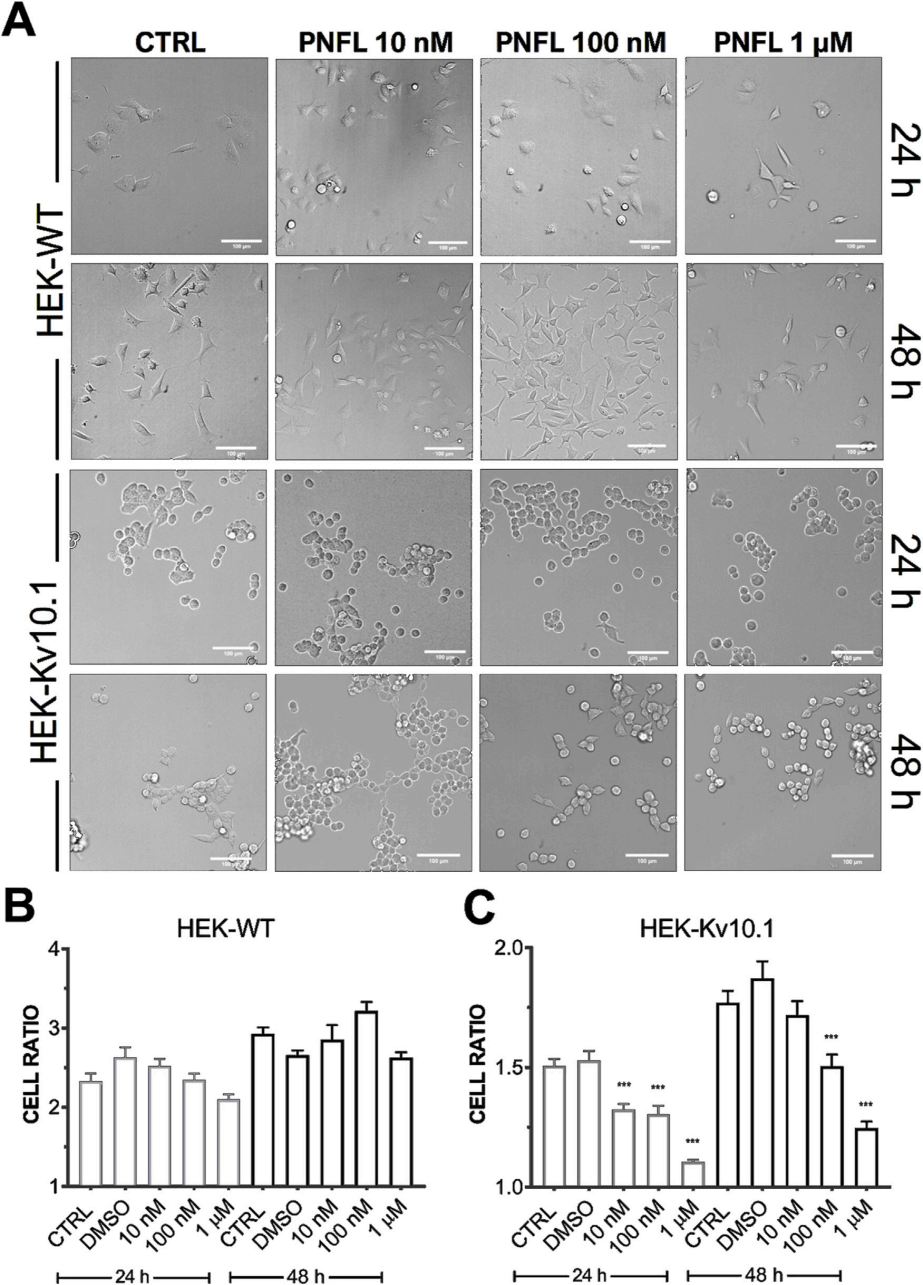
Penfluridol alters the cellular morphology of cells expressing the Kv10.1 channel. **(A)** Representative cell morphology images per line in control and after 24 and 48 h PNFL treatment. **(B,C)** Summary of the morphological cell ratio quantification of HEK-WT and HEK-Kv10.1, respectively, in control and the presence of PNFL. *** for a P < 0.001, one-way ANOVA.

Although PNFL did not significantly affect HEK-Kv10.1 cell viability, it was notable that PNFL affected cell morphology. The morphological features of HEK-WT and HEK-Kv10.1 cells were examined using light microscopy and monitored after 24 and 48 h of PNFL (10 nM, 100 nM, and 1 µM) treatment. Major and minor cell axes were measured, and the morphological cell ratio (MCR; see methods) was calculated to analyze morphological changes quantitatively. HEK-WT cells exposed to different PNFL concentrations did not show changes in morphology after 24 h or 48 h compared to the control situation (Figures 5A,B). In contrast, HEK-Kv10.1 cells changed their shape to a more rounded morphology after 24 and 48 h of PNFL treatment (Figures 5A,C). Neither type of cell modified its morphology in the presence of the vehicle (0.1%).

## 4 Discussion

*De novo* drug discovery is a slow, expensive, and high-risk process. However, this drawback can be reasonably addressed by repurposing FDA-approved drugs to extend their therapeutic properties in new clinical areas, such as oncology (DiMasi et al., 2016; Ashraf-Uz-Zaman et al., 2018; Pantziarka et al., 2020; Jain et al., 2023). One such candidate is PNFL, an antipsychotic drug with reported anticancer effects, although its underlying mechanisms remain poorly understood (Wu et al., 2014; Tuan and Lee, 2019). In this study, we investigated the effects of PNFL on the functional activity and associated cellular behavior of the oncogenic channel Kv10.1. Our findings demonstrate that PNFL is a potent functional inhibitor of Kv10.1, suggesting that this inhibition may contribute to the reduction of key cancer-related processes, such as cell migration.

Although PNFL has been shown to reduce hallmark features of cancer, including tumor growth, metastasis, and angiogenesis in various cancer types (Wu et al., 2014; Ranjan and Srivastava, 2016; Ranjan et al., 2016; 2017; Xue et al., 2020; Lai et al., 2022), the proposed mechanisms vary widely and include effects on ion channels. PNFL, as well as other derivatives of diphenylbutylpiperidine, such as pimozide and I-cis-diltiazem, were shown to block an AMP-sensitive potassium channel, with PNFL exhibiting notable potency (Gomora and Enyeart, 1999). PNFL has also been described as a calcium channel blocker (Enyeart et al., 1993; Zamponi et al., 1996; Santi et al., 2002; Dziegielewska et al., 2014). Nevertheless, the Kv10.1 channel has not yet been reported as a molecular target of PNFL.

Here, membrane potential fluorescence assays showed that PNFL significantly reduced the depolarization-induced fluorescence signal in HEK-Kv10.1 cells, with greater efficacy than the well-characterized Kv10.1 inhibitor loperamide. This finding was corroborated by whole-cell patch-clamp recordings, where PNFL nearly abolished Kv10.1 currents in a dose-dependent manner, with an IC_50_ of 2.7 µM. Remarkably, no effect was observed in HEK-WT cells in the presence of PNFL. Furthermore, the inhibitory effect of PNFL persisted after washout (data not shown), suggesting an irreversible block. A similar effect has also been reported on T-type calcium channels (Santi et al., 2002). While this could reflect a high-affinity interaction between PNFL and Kv10.1 channels, we cannot exclude the possibility that PNFL induces changes in the constitutive internalization of the channel (Kohl et al., 2011; Herrmann et al., 2012). Further experiments will be needed to distinguish between these mechanisms. The fitted Hill coefficient may indicate negative cooperativity, whereby the binding of one PNFL molecule reduces the affinity of subsequent ones, or may reflect multiple non-equivalent binding sites on Kv10.1. The nearly complete and irreversible inhibition observed at high concentrations (100 µM) supports the possibility of high-affinity interactions or conformational changes that persist after drug removal. Further studies are needed to determine whether this effect results from direct channel binding or modulation through intracellular pathways.

The inhibitory action of PNFL profoundly impacted the migration behavior of cells expressing Kv10.1, but not that of wild-type HEK293 cells. This finding suggests that PNFL’s anti-migratory effect is specifically dependent on Kv10.1 activity, reinforcing the idea of Kv10.1 as a viable therapeutic target in metastatic processes where it is overexpressed. These findings are consistent with previous reports linking Kv10.1 activity to oncogenic processes such as proliferation, angiogenesis, and migration (Luis et al., 2022a). Potassium channels are known to influence cell migration (Schwab et al., 2008), and Kv10.1, in particular, plays an active role in this process (Hammadi et al., 2012; Valdés-Abadía et al., 2019). In MDA-MB-231 breast cancer cells, Kv10.1 silencing reduces cell migration through mechanisms involving alterations in resting membrane potential and calcium influx via Orai1 (Hammadi et al., 2012). A similar mechanism may underlie the reduced migration observed in our HEK-Kv10.1 cell model, which expresses Orai1 (El Boustany et al., 2010). Furthermore, pharmacological modulation of Kv10.1 has been implicated in modifying the resting membrane potential (Gómez-Herrera et al., 2023).

Significantly, PNFL did not affect overall cell viability in either HEK-WT or HEK-Kv10.1 cells, indicating that the observed reduction in migration is not due to general cytotoxicity but to specific Kv10.1-related mechanisms. This finding enhances the profile of PNFL as a selective and potentially safe compound for therapeutic intervention, although further *in vivo* studies are needed to validate this profile. Interestingly, despite the lack of cytotoxicity, PNFL induced notable morphological changes in HEK-Kv10.1 cells, which adopted a more rounded shape after treatment. As cell morphology is closely associated with cytoskeletal organization and migratory capacity (Seetharaman and Etienne-Manneville, 2020), this change may be functionally linked to the observed reduction in cell migration. These effects suggest that PNFL might influence additional signaling pathways associated with Kv10.1, such as those regulating cytoskeleton dynamics, cell adhesion, or polarity.

The morphology changes induced by PNFL in HEK-Kv10.1 cells, absent in the wild-type line, further support a role for Kv10.1 in regulating cell shape, possibly independent of integrin-related pathways previously associated with PNFL (Toral et al., 2007; Ranjan et al., 2016). Ion channels are known to interact extensively with cytoskeletal elements (Janmey, 1998; Rodat-Despoix et al., 2021). In the case of Kv10.1, several interactions with cytoskeleton elements (Camacho et al., 2000; Toral et al., 2007; Herrmann et al., 2012; Movsisyan and Pardo, 2020) may be involved in the acquisition of the round-shape phenotype by HEK-Kv10.1 cells in the presence of PNFL. A similar role has been described for the Kv10.2 channel; the loss-of-function of this channel modified the cell shape of primary keratinocytes in culture (Galán-Vidal et al., 2022).

This study has some limitations. Although our cell models provide a controlled environment for assessing Kv10.1 function, extrapolation to *in vivo* tumor models should be approached with caution. Future studies are required to determine whether PNFL induces similar effects in cancer cell lines that endogenously overexpress Kv10.1 and to evaluate its efficacy in animal models. Additionally, the precise mechanism by which PNFL modulates Kv10.1, whether through direct binding or upstream/downstream signaling pathways, remains to be fully elucidated.

In conclusion, our results identify PNFL as a potent negative modulator of Kv10.1 channel function, with downstream effects on cell migration and morphology. These findings open new avenues for future studies of penfluridol on cancer cell lines that overexpress Kv10.1.

## Data Availability

The raw data supporting the conclusions of this article will be made available by the authors, without undue reservation.

## Supplementary material

SUPPLEMENTARY FIGURE 1 Penfluridol does not affect the activity of HEK-WT cells. **(A)** Fluorescence membrane potential recordings in HEK-WT cells under control conditions and after treatment with PNFL (100 µM). Solid lines represent the mean, and faded colors indicate the SEM. **(B)** Quantification of fluorescence amplitude in HEK-WT cells under control conditions and with PNFL. Bars represent mean ± SEM from three independent experiments. **(C)** Representative current traces recorded from HEK-WT cells under control conditions (blue), in the presence of PNFL (100 μM, green), and after washout (gray). **(D)** Current-voltage (I–V) relationship under the same three conditions (n = 5).
