# Supplementary figures and images for: Inhibition of the oncogenic channel Kv10.1 by the antipsychotic drug penfluridol
Source: Front Pharmacol. 2025 Sep 3;16:1655406. doi: 10.3389/fphar.2025.1655406 (PMC12440886; doi:10.3389/fphar.2025.1655406)

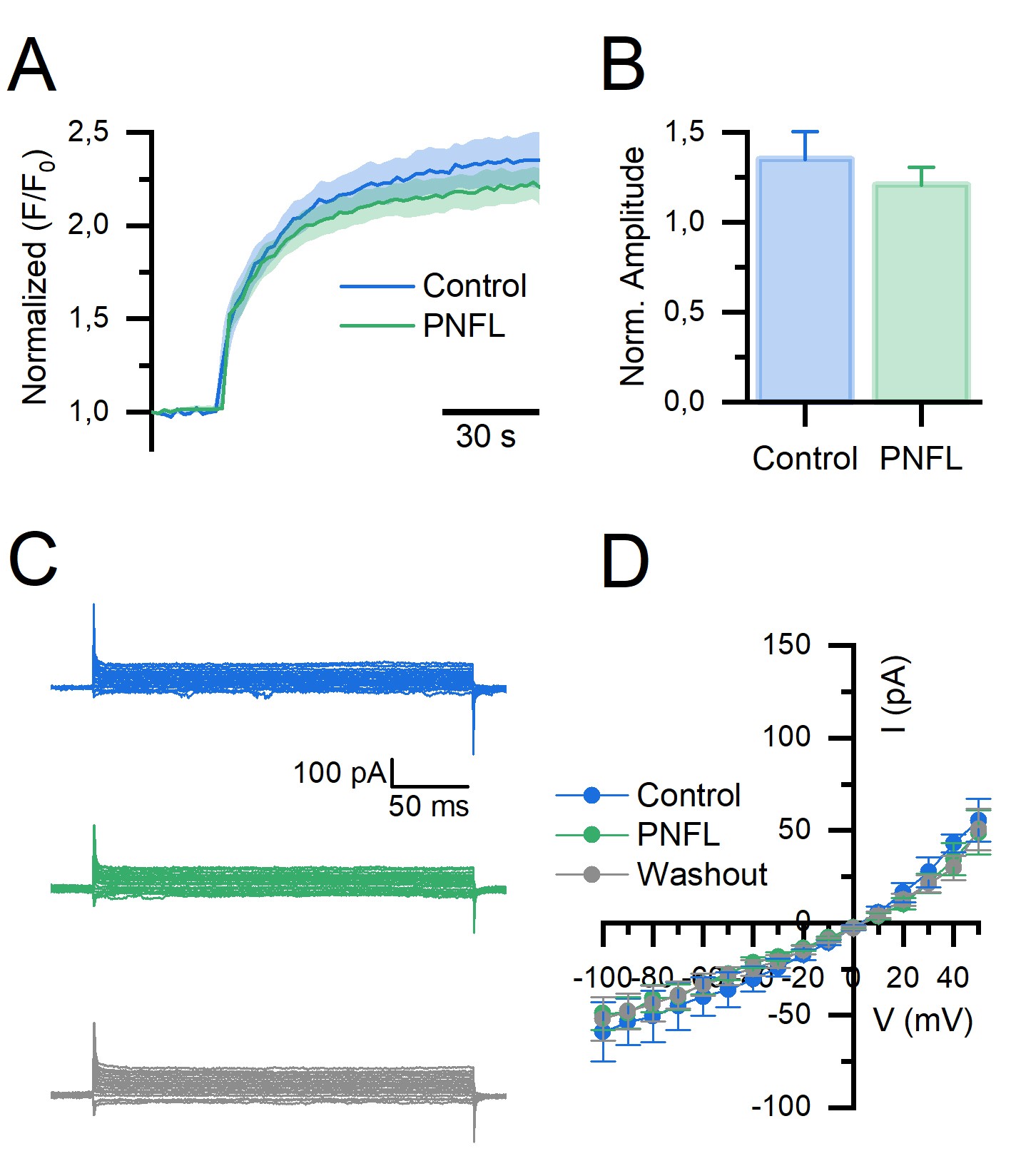

Supplement: Supplementary file 1 [file Image1.jpeg]
